# Ureteroscopy at ambulatory surgery centers can be safe for select high-risk patients

**DOI:** 10.1007/s00345-025-05632-2

**Published:** 2025-04-22

**Authors:** Ryan Cook, Andrewe L. Baca, Alexander Small, Carina Himes, Dima Raskolnikov

**Affiliations:** 1 Albert Einstein College of Medicine, 1945 Eastchester Road, Bronx, NY USA; 2 Department of Urology, Albert Einstein College of Medicine, 1250 Waters Place, Tower One, PH, Bronx, NY 10461 USA; 3 Department of Anesthesiology, Albert Einstein college of Medicine, 111 East 210 Street, Bronx, NY 10467 USA

**Keywords:** Outpatient ureteroscopy, Ambulatory surgery center, Emergent transfer, High-risk, Kidney stones

## Abstract

**Purpose:**

To determine whether unhealthy patients undergoing ureteroscopy at an ambulatory surgery center are at high risk of emergency post-operative transfer to an inpatient setting.

**Methods:**

Records of 100 consecutive high-risk patients undergoing URS at a free-standing ambulatory surgery center (ASC) were reviewed. Patients were considered high-risk if their American Society of Anesthesiologists score (ASA) was ≥ 3. Perioperative risk was assessed using the Charlson Comorbidity Index (CCI) and the 5-factor Modified Frailty Index (mFI-5). Post-operative complications requiring emergent inpatient transfer on the day of surgery and complications within 30-days were recorded.

**Results:**

The median age for patients was 61.4 years (IQR: 54.7–68.4) and median BMI was 31.0 (IQR: 26.6–37.2). Common chronic illnesses were hypertension (74%), COPD (44%), diabetes (40%), and peripheral vascular disease (14%). All patients were considered high-risk by ASA score. By CCI, 28% of patients were high-risk, 23% were significant risk and 49% were mild risk, with median score of 3 (IQR 1–5). By mFI-5, 20% of patients were high-risk, with median score of 2 (IQR 1–2). No patient required emergent transfer on the day of surgery. Within 30 days postoperatively, 15 patients presented to the Emergency Department (ED) and 5 were admitted. mFI-5 and CCI were not predictive of ED visits.

**Conclusion:**

Selected high-risk patients with unfavorable ASA, mFI-5, and CCI undergoing URS within an ASC are unlikely to require emergent, post-operative transfer to an inpatient facility.

## Introduction

The high cost of inpatient care has led to a shift towards performing procedures in Ambulatory Surgical Centers (ASCs), which offer greater efficiency, lower costs, and in some cases, decreased post-operative complications [1–5]. Ureteroscopy (URS) is particularly well-suited for ASCs because it is a relatively low risk procedure and nearly always ambulatory [6–8]. 

Despite the increasing prevalence of URS in ASCs, there are no established guidelines to help urologists determine which patients are acceptable candidates based on medical risk. This issue is more pronounced in resource-limited hospitals, which must balance economic considerations while treating medically disadvantaged, often high-risk patients [9]. Performing URS on patients with a higher burden of systemic illnesses may increase the risk for anesthetic complications, potentially requiring transfer to an inpatient setting—a time-sensitive process necessitating ambulance transport when done at an ASC [10]. Conversely, given the inherently low-risk of URS, it is also plausible that unhealthy patients can undergo URS in an ASC without compromising safety.

Previous studies have demonstrated the safety of performing URS in ASCs among healthy, low-risk patients [8, 11]. The purpose of this study was to evaluate the safety of URS in ASCs among high-risk patients with significant medical comorbidities. These findings aim to guide urologists in making informed decisions about patient selection for URS in ASCs. Ultimately, this work has the potential to improve surgical care accessibility and safety for high-risk patients in resource-limited settings.

## Methods

Billing records were retrospectively reviewed for all high-risk patients who underwent outpatient URS for the treatment of kidney or ureteral stones at an ASC between February 2022 and March 2023. This ASC is part of an academic health care system in an urban setting. Patients were included if their American Society of Anesthesiology (ASA) score was ≥ 3, as this score suggests the presence of severe systemic disease [12]. Patients with an ASA score of 1 or 2 were excluded from this study. An ASA score of 1 indicates a healthy patient with no significant medical conditions, while an ASA score of 2 reflects a patient with mild systemic disease that does not significantly impact daily function [12]. All patients underwent general anesthesia followed by flexible or semirigid ureteroscopy, with specific surgical technique dictated by surgeon preference. Variables such as use of ureteral access sheath, irrigation type, laser modality, and stent placement were not standardized.

Patient charts were reviewed for immediate peri-operative complications requiring emergent hospital transfer, as well as any complications within 30 days of surgery requiring Emergency Department (ED) visits. Patient demographics, comorbidities, and operative variables were recorded. Preoperative risk was assessed using the classic Charlson Comorbidity Index (CCI) and the 5-factor Modified Frailty Index (mFI-5), as has been done previously [13, 14]. 

To calculate CCI, data was collected with respect to 19 medical conditions, where each condition has a predetermined weighted score ranging from 1 to 6 according to its hazard ratio for 1-year mortality [15]. Per classic CCI guidelines, age was not directly evaluated. The mFI-5 score was calculated by adding one point for each of: diabetes, hypertension, COPD, congestive heart failure, and functionally dependent health status [14]. CCI scores were categorized as mild (0–2), significant (3–4), or high (5+) and mFI-5 scores were categorized as mild (0–3) or high (3+) [16, 17]. 

Data was assessed using descriptive and bivariate inferential statistics. Continuous variables were analyzed using Wilcoxon rank-sum test and are presented as means with associated standard deviations. Discrete variables were assessed using chi-squared analysis or Fischer’s exact test. All statistical analyses were performed using SPSS, version 28 (IBM Corp., Armonk, NY, USA).

## Results

We reviewed a total of 378 patient records who underwent URS. 25 were excluded for having an ASA of 1 and 253 were excluded for having an ASA of 2. This yielded a final study cohort of 100 patients, all of whom had an ASA of 3 at the time of URS (Table 1). All 100 patients were included in the final analysis, including 3 patients that were lost to follow up, despite multiple attempts to contact them via voicemail and mailed letters. The median age was 61.4 years (IQR: 54.7–68.4) and the median BMI was 31.0 (IQR: 26.6–37.2). Regarding race, 26% of patients identified as Black, 22% as White, 2% as Asian, and 50% as Other. Regarding ethnicity, 50% of patients identified as Hispanic. The most common chronic illnesses were hypertension (74%), COPD (44%), diabetes (40%), peripheral vascular disease (14%), and liver disease (13%). Patients had a median of 2 stones (IQR: 1–3), with a median greatest stone diameter of 8.6 mm (IQR: 6.0–11.0). Patients had a median procedure time of 99.8 min (IQR: 78.0–122.0). Pre-operative urinalysis was negative for nitrite in 91 patients and positive in 9 patients. Pre-operative urine culture was defined as positive at > 50,000 colony forming units per milliliter [18], and was positive in 10 patients (10%). 5 patients were pre-treated with oral antibiotics, while the other 5 were not based on shared decision making in the setting of asymptomatic bacteriuria. 51 patients had significant or high-risk CCI scores (23% and 28%, respectively), with median scores of 3 (IQR 1–5) (Table 2). Additionally, 20 patients (20%) were classified as high-risk by the mFI-5, with median score of 2 (IQR 1–2).

**Table 1:** Patient demographics, comorbidities, risk scores, stone characteristics, and post-operative outcomes

*n*	100
**Gender (Female)**	59
**Age**	60.0 (54.7–68.4)
**Race**	
Black	26
White	22
Asian	2
Other	50
**Ethnicity (Hispanic)**	50
**BMI**	31.8 (26.6–37.2)
**ASA Score of 3 or higher**	100
**Comorbidities**	
Hypertension	74
COPD	44
Diabetes	40
Peripheral Vascular Disease	14
Liver Disease	13
**mfI-5**	
Low Risk	80
High Risk	20
**CCI**	
Mild Risk	49
Significant Risk	23
High Risk	28
**Number of Stones Prior to URS**	2.0 (1.0–3.0)
**Largest stone diameter (millimeters)**	8.6 (6.0–11.0)
**Procedure time (minutes)**	99.8 (78.0–122.0)
**Immediate hospital transfers following URS**	0
**Emergency Room Visits Within 30 days of surgery**	15
**Hospital admissions following ED visit**	5

Values are n, or median (Interquartile Range). Abbreviations: ED, emergency department; BMI, body mass index; ASA, American Society of Anesthesiologists; COPD, chronic obstructive pulmonary disease; mFI-5, 5-factor Modified Frailty Index; CCI, Charlson Comorbidity Index; URS, ureteroscopy

**Table 2:** Characteristics of patients who did and did not visit the emergency department

	No ED Visit within 30 days (*n* = 85)	ED Visit within 30 days (*n* = 15)	*p*-value
Gender (Female)	49	10	0.50
Age	60.4 (55.0–68.6)	57.7 (44.3–68.5)	0.47
Race			0.10
Black	24	2	
White	16	6	
Asian	1	1	
Other	44	6	
Ethnicity (Hispanic)	44	6	0.28
BMI	31.8 (26.6–37.1)	31.9 (23.7–39.3)	0.91
Comorbidities			
Hypertension	62	12	0.55
COPD	37	7	0.82
Diabetes	37	3	0.07
Peripheral Vascular Disease	13	1	0.39
Liver Disease	10	3	0.38
mFI-5			0.29
Low Risk	66	14	
High Risk	19	1	
CCI			0.87
Mild Risk	42	7	
Significant Risk	20	3	
High Risk	23	5	
Reason for ED Visit within 30 days			
UTI	–	4	
Non-UTI, Urologic Complaint	–	8	
Non-Urologic Complaint	–	3	
Symptoms at Follow-Up Visit			
Abdominal Pain	10	1	
Urinary symptoms	6	0	
Back or Flank Pain	4	0	
Bladder spasm	1	0	
Fever	1	1	
Number of Stones Prior to URS	2.0 (1.0–3.0)	1.9 (1.0–3.0)	0.66
Largest Stone Diameter (millimeters)	8.4 (6.0–11.0)	9.4 (6.0–13.0)	0.44
Procedure time (minutes)	98.6 (76.5–123.2)	106.0 (90.0–121.0)	0.20

Values are n, or median (Interquartile Range). Abbreviations: ED, emergency department; BMI, body mass index; ASA, American Society of Anesthesiologists; COPD, chronic obstructive pulmonary disease; mFI-5, 5-factor Modified Frailty Index; CCI, Charlson Comorbidity Index; UTI, urinary tract infection; URS, ureteroscopy

Following URS, no patients required emergency transfer from the PACU to an inpatient facility. However, within 30 days postoperatively, 15 patients (15%) presented to the ED and 5 patients (5%) were subsequently admitted. Of the 15 patients who presented to the ED after URS, 3 presented 0–2 days after, 5 presented 3–7 days after, 4 presented 8–14 days after, and 3 presented 15–30 days after. The median post-op day was 7 (IQR 3–14). Among those presenting to the ED, 12 reported urological symptoms. Common complaints included abdominal pain (8 patients), flank pain (6 patients), and hematuria (6 patients). 4 of these 12 patients were diagnosed with urinary tract infections (UTIs), confirmed by urine cultures that met the preoperative definition of > 50,000 colony-forming units. The remaining 8 patients reported stent-related symptoms initially suspected to be UTIs, though their urine cultures were ultimately negative. CCI, mFI-5, procedure time, and stone burden were all not found to be significantly associated with risk of ED presentation after surgery.

Among the 85 patients who did not require an ED visit, 19 (22%) had documented mild post-operative complications based on self-report of symptoms described in clinic notes. Of these patients, notable complaints included abdominal pain (10 patients), transient urinary symptoms like hematuria, dysuria, or urgency (6 patients), back or flank pain (4 patients), bladder spasm (1 patient), and transient fever (1 patient). Stents had been placed in 13 of these patients, and their symptoms were deemed consistent with expected stent-related discomfort. The symptoms of the 6 patients without stents were attributed to normal post-operative recovery. All 19 patients were managed conservatively with increased fluid intake and over the counter medications. None of these patients required escalation of care.

Of the 15 patients who presented to the ED within one month of URS, 13 had not attended a post-operative clinic visit prior to their ED presentation, while 2 attended follow-up before going to the ED. One of these patients was advised to seek emergency care after reporting fever at their follow-up appointment, while the other presented with abdominal pain, which was later attributed to stent-related discomfort.

## Discussion

Among 100 high-risk patients, there were no emergency transfers to an inpatient facility for higher level post-operative care. When considering the patients who required ED visits following URS, neither CCI nor mFI-5 was predictive of their return for medical care. Collectively, this study demonstrates that high-risk patients, as indicated by unfavorable ASA, mFI-5, and CCI scores, may safely undergo URS within an ASC.

The current expected rate of immediate transfer to the inpatient setting following outpatient URS is poorly understood due to limited data on this subject [19]. Tan et al. report an immediate admission rate of 3.9% between 1998 and 2008, and Mittakanti and colleagues report a 2.2% admission rate within a 7-day window from 2010 to 2012 [10, 20]. The observed findings provide a comparable admission rate even after stratification by health status and operative risk, suggesting that underlying health risk may not significantly impact the likelihood of admission after URS. In turn, these findings indicate that patient selection for outpatient URS may safely include traditionally high-risk individuals. It should be noted that that the results of Tan et al. are appreciably dated and Mittakanti et al. did not evaluate for immediate hospital admission, thereby limiting further conclusions regarding which URS cases are appropriate for ASCs.

In the 30 days following URS, 15 (15%) of patients in the present study visited the ED and 5 (5%) were admitted. Despite a focus on exclusively high-risk patients, these outcomes mirror those seen in studies with both low and high-risk patients [19–21]. For example, Du et al. reviewed a healthier cohort of patients undergoing URS in an ASC, characterized by only 19% of patients with a CCI ≥ 2 and only 35% with an ASA ≥ 3, as opposed to 100% ASA ≥ 3 in the present study [11]. Despite this lower risk profile, 14.6% of patients presented to the ED and 5.1% of patients were admitted in the 30 days following surgery, further suggesting that high-risk individuals can safely undergo outpatient URS.

Although all patients were classified as high-risk based on ASA, only 28% were deemed high-risk when evaluated by the CCI and 20% by the mFI-5, according to thresholds established in prior studies [16, 17]. This discrepancy highlights the potential differences in what each scoring system evaluates and prioritizes. The ASA score is a more general measure of a patient’s overall health, relying heavily on the subjective assessment of the clinician [22]. In contrast, the CCI and mFI-5 provide more objective, quantifiable assessments of comorbidities and frailty, respectively, which may not align perfectly with the broader scope of the ASA classification. Nonetheless, prior studies of patients undergoing urologic surgery define high-risk patients to be those with a CCI threshold of ≥ 2 and mFI-5 threshold of ≥ 1 [23, 24]. Using this more conservative definition of high-risk, 45% of patients in the present cohort would have been high-risk by CCI ≥ 2 and 88% were high-risk by mFI-5 ≥ 1. These findings suggest that the ASA, CCI, and mFI-5 may capture different aspects of perioperative risk, and reliance on a single metric may overlook important nuances in patient risk stratification.

This study has limitations that moderate its ability to inform specific policy changes. First, the retrospective design introduces the possibility of confounding. Although all study patients were high-risk, urologists proactively selected the patients to have surgery within an ASC, and anesthesiologists agreed with this assessment on the day of surgery. Second, variability in provider documentation, where some clinicians explicitly note symptoms while others broadly classify findings as “normal” or “abnormal” without further elaboration, may contribute to a general underreporting of symptoms at post-operative visits. Third, the sample size is small, and a larger dataset may reveal that certain patients at particularly high risk do ultimately require peri-operative transfer. Fourth, we retrospectively collected data on comorbidities to inform CCI and mFI-5 scoring. While all patients at least had detailed perioperative assessments by an anesthesiologist, it’s possible that existing comorbidities were not fully documented. Such an omission would in fact make the patients appear to be healthier in our study than they actually were. Lastly, there is likely unmeasured selection bias inherent to this data. Despite these limitations, such findings still form a useful first step in designing future prospective studies that evaluate the safety of URS in an ASC for higher risk patients.

Further research is needed to identify specific criteria that can help risk-stratify patients for URS and optimize the use of ASCs. For example, ASCs typically limit URS to patients with an ASA of 1 or 2, but the present findings suggest this may be an overly conservative approach [7]. Future analysis should address this by evaluating a more robust patient cohort with continued focus on high-risk patients. Going forward, physicians should continue to evaluate each patient on a case by case basis when selecting the setting for URS, with particular scrutiny of factors like morbid obesity, history of kidney stone-related admissions, and the need for bilateral URS [20, 25]. These factors have been identified in the literature as potential predictors of immediate hospital admission following outpatient URS and therefore warrant additional consideration [20, 25]. Real-time assessment of the specific factors influencing these decisions will be essential before clear guidelines can be established. Although challenges remain in defining this standardized criteria, expanding access to outpatient URS has the potential to improve surgical efficiency, reduce costs, and ultimately enhance patient outcomes [7, 8]. 

## Conclusion

Selected high-risk patients with unfavorable ASA, mFI-5, and CCI scores may safely undergo ureteroscopy within an ambulatory surgery center. Despite these high-risk features, such patients are unlikely to require emergency transfer from PACU to an ED. Further research is needed to risk stratify the highest risk patients who may benefit from surgery in the hospital setting.

## Data Availability

No datasets were generated or analysed during the current study.
